# Strengthening Interpersonal Relationships in Maternal and Child Health Care in Rural Tanzania: Protocol for a Human-Centered Design Intervention

**DOI:** 10.2196/37947

**Published:** 2022-07-07

**Authors:** Kahabi Isangula, Constance Shumba, Eunice S Pallangyo, Columba Mbekenga, Eunice Ndirangu-Mugo

**Affiliations:** 1 School of Nursing and Midwifery Aga Khan University Dar Es Salaam United Republic of Tanzania; 2 School of Nursing and Midwifery Aga Khan University Nairobi Kenya

**Keywords:** human-centered design, user-centred design, human-centred design, provider-patient relationship, nurse-client relationship, nurse, nursing, maternal and child health, maternal, maternity, mother, child, primary health care, primary care, rural, Tanzania, Africa, community-based, focus group, co-design, prototype, validation, nurse-patient, provider-client

## Abstract

**Background:**

Evidence indicates that clients’ dissatisfaction with providers’ competencies within maternal and child health (MCH) continues to impact trust in formal health care systems, service uptake, continuity with care, and MCH outcomes. A major problem with existing interventions is the failure to address all the complexities of provider-client relationships necessitating targeted, contextualized, innovative solutions that place providers and clients at the forefront as agents of change in optimizing intervention design and implementation. To improve the provider-client relationship, the Aga Khan University is piloting a human-centered design (HCD) intervention where MCH nurses and clients are invited to partner with researchers in the intervention design and evaluation process.

**Objective:**

The objective of this research is to co-design an intervention package (prototype) for improving nurse-client relationships in the rural Shinyanga region of Tanzania using a series of iterative HCD steps, involving key stakeholders to tailor solutions for complex problems impacting provider-client interactions in MCH care.

**Methods:**

The following 5-step HCD approach will be implemented: (1) community-driven discovery through qualitative descriptive research methods using focus group discussions and key informant interviews; (2) co-design of an intervention package through consultative ideation and cocreation meetings with nurses, clients, and other stakeholders; (3) prototype validation through qualitative insight gathering using focus group discussions; (4) refinement and adaptation meeting; and (5) documentation and sharing of lessons learned before the final prototype is tested and validated in a broader community.

**Results:**

A prototype characterized by a package of interventions for improving nurse-client relationships in MCH care in rural contexts is expected to be developed from the co-design process.

**Conclusions:**

An HCD approach provides a novel entry point for strengthening provider-client relationships, where clients are invited to partner with providers in the design of acceptable and feasible interventions.

**International Registered Report Identifier (IRRID):**

DERR1-10.2196/37947

## Introduction

Nurses and midwives form a critical component of maternal and child health (MCH) services globally. They play a vital role in the delivery of primary health care services related to pregnancy monitoring, delivery, and postnatal care for women and newborns around the world [1-4]. In sub-Saharan Africa, nurses and midwives are often respected members of the community and provide advice and evidence-based information on a range of health issues, including care of newborns and young children [2-4]. With sufficient, well-supported, and competent nurses and midwives, 83% of maternal deaths, stillbirths, and neonatal deaths could be prevented [5-7]. Competent nurses and midwives have the potential to increase client service uptake and continuity, and consequently improve health outcomes, such as increased breastfeeding initiation and duration, and reductions in cesarean sections, maternal infections, postpartum hemorrhage, and preterm births [8]. Literature suggests that investing in nurses and midwives has the potential to yield a 16-fold return on investments resulting from improved MCH outcomes [9].

Despite the critical role of nurses and midwives, there has been increasing client dissatisfaction with the interpersonal and perceived technical competencies of nurses and midwives within MCH care in recent years [10-19]. Perceived technical incompetence associated with skills, reliability, assurance, confidentiality, and patient engagement; behavioral incompetence involving demeanor, attitudes, and empathy; communication skills and language; and violation of client rights and respect continue to obscure the positive value of nurses and midwives in the delivery of MCH interventions in Tanzania and other settings [10-21]. Recent studies indicate that clients’ dissatisfaction with nurses’ interpersonal and technical aspects of care continues to erode client trust in the formal health care system, service uptake, continuity, and MCH outcomes [19-23].

To address clients’ dissatisfaction, health care service governance instruments including policies, client service charters, health facility governance committees, complaints mechanisms, and professional bodies have been emphasized in both high- and low-income settings; however, their effectiveness is not well established. Consequently, political interventions such as employment termination and labeling of nurses as “bad, lazy, and incompetent” are the current actions taken for addressing this complex problem, creating tension between clients and nurses as well as contributing to the poor morale of providers [24,25]. Competence-based interventions focusing on provider communication skills, patient-centered care, patient literacy, information seeking, participation, and questioning skills are often implemented erratically, yielding unsatisfactory results. A major challenge with existing interventions documented in the literature is the failure to address all the complexities of nurse-client relationships along the continuum of MCH care. Patients’ socioeconomic fragility, literacy, and behaviors coupled with providers’ poor interpersonal skills and health system challenges add to the complexity of nurse-client relationships. This complexity necessitates targeted contextualized and innovative solutions that place nurses and clients at the forefront as agents of change in optimizing the design and implementation of interventions [26].

Rather than replicating existing interventions that may not be contextually applicable, new and innovative interventions to improve the provision of high-quality and satisfactory care are needed within resource-constrained settings such as rural Tanzania [26-32]. If embraced, a stepwise incremental process from intervention design to evaluation of effectiveness could offer flexibility in problem-solving while using a standardized process that has the potential to be applied in diverse settings. It is within this context that the Aga Khan University (AKU) is piloting a human-centered design (HCD) intervention in rural Tanzania, where nurses and MCH clients are invited to partner in the intervention design and evaluation process to deepen understanding of and address the identified challenges. Abookire et al [31] consider HCD as “an innovative approach to problem-solving that leverages insights from the end users of new products, services, and experiences to develop best-fit solutions that are rapidly prototyped and iteratively refined.” HCD is considered to facilitate improvements in client, provider, and community satisfaction, as well as increased efficiency and collaboration in public health intervention development and implementation process [30-32]. Furthermore, HCD may result in more successful and sustainable interventions compared to traditional problem-solving approaches in health care and public health [31]. Melles et al [30] recently proposed that the implementation of HCD in health care needs to focus on developing an understanding of the people facing a particular barrier and their needs and engaging them as stakeholders throughout the design process. The HCD approach also embraces a system-wide outlook by considering interactions of factors at different levels and harmonizing individual interests to form collective interests when developing solutions. Therefore, we aim to co-design an intervention package (prototype) for improving nurse-client relationships in the Shinyanga region of rural Tanzania. We will use a series of iterative HCD steps involving key stakeholders to contextualize solutions for complex problems impacting interactions in MCH care. We hypothesize that the emerging prototype will have high potential in improving nurse-client relationships, thereby leading to increased client satisfaction, MCH service uptake, and service continuity in rural communities.

## Methods

### Design

A 5-step HCD approach will be employed as an investigative framework to co-design interventions for improving nurse-client relationships using qualitative descriptive design with focus group discussions (FGDs), key informant interviews (KIIs), and consultative meetings. This approach was deemed appropriate to answer two key questions: (1) What are the drivers of poor nurse-client relationships in MCH care? and (2) What is/are the best intervention codeveloped by nurses and clients for strengthening nurse-client relationships to address these drivers considering feasibility and acceptability? A qualitative descriptive approach is appropriate for this inquiry as it aims to develop understanding and describe nurse-client relationships without testing an existing theory [33]. This approach offers an effective way of gaining a deep and rich understanding of nurse and client perceptions and experiences in the chosen context, as this may differ from other contexts in terms of culture, expectations, and resources within health care settings. The qualitative descriptive design also allows us to acknowledge the subjectivity of nurses and clients as well as researcher experiences of nurse-client relationships and the research process to collect data in a natural setting. Furthermore, by listening to the descriptions of nurses and clients, we could learn from these experiences of MCH care and use “…this knowledge to influence interventions design” using the HCD process and generate research findings of “specific relevance to practitioners and policy makers” to improve MCH care [33].

As an investigative framework for this study, HCD is a problem-solving approach that uses a series of iterative and often nonlinear steps to tailor solutions for complex problems [26-32]. Although similar to other participatory research frameworks in their inclusion of end-user feedback throughout the research process, HCD differs in its endeavor toward empathy, a deep understanding of the motivations and desires that govern human behavior, as the inspiration and core of intervention development [26]. In the HCD approach, end users are invited to partner in the design and evaluation process to better understand, meet, and address the identified challenges. In a low-resource complex setting where the drivers of poor nurse-client relationships may differ significantly from well-resourced settings, these key principles of HCD can be leveraged to optimize intervention development and implementation.

### Settings

This study will be conducted in Shinyanga, a region located in Lake Zone and predominantly inhabited by Bantus. Isangula [23] offers a detailed description of the region. Briefly, Shinyanga falls within the low-income category of the regions in Tanzania. It is administratively divided into 5 districts: Shinyanga Municipal Council (MC), Shinyanga District Council (DC), Kishapu DC, Kahama MC, and Kahama DC. The rationale for choosing Shinyanga is twofold. First, ethnically, the region is predominantly inhabited by Sukuma, who share a range of sociocultural beliefs and practices with minimal diversity. Due to its near homogeneity, the region is a perfect exemplar of many other rural regions of Tanzania. Second, despite a number of capacity-building interventions, local data indicate enormous concerns about poor nurse-client relationships in MCH care [23]. Within the Shinyanga region, Shinyanga MC was purposefully selected because patients in this district have greater access to both the formal health care system (mostly public and few private and faith-based facilities) and traditional care [23].

By focusing on the Shinyanga region, we will embrace the importance of deepening our understanding of the unique barriers to nurse-client relationships in this setting and aim to provide a context-specific intervention model that is applicable in this and similar contexts. The UK Medical Research Council’s framework for the development and evaluation of complex interventions underlines the crucial role of the context in the adaptation and implementation of interventions [34]. The region, like many other parts of Tanzania and sub-Saharan Africa, has a wide range of rural and urban populations with varying socioeconomic statuses including highly marginalized populations with immense potential to positively impact population health. However, given the contextual differences within Tanzania and across Africa, the prototype generated may differ but still provide an applicable and exemplary model for feasibility testing and adaptation in diverse settings.

The concerns of poor nurse-client relationships in MCH documented in Shinyanga have been previously documented in other rural regions of Tanzania and Africa. This means the prototype developed in Shinyanga may be feasible in other rural regions of Tanzania and Africa with some minor adaptations. However, further testing and refinement of the prototype during the feasibility study may offer more insights into the feasibility of the prototype in other regions of Tanzania and beyond.

### Study Population, Sample Size, Sampling, and Data Collection

The 5-step HCD process is envisaged below (Multimedia Appendix 1).

#### Step 1. Community-Driven Discovery Inquiry

A combination of qualitative research methodologies will be employed to explore community and individual perspectives. A minimum of 8 FGDs and 10 KIIs will be conducted with purposefully selected nurses and midwives, women attending MCH services, and administrators using a semistructured interview guide in the Swahili language. We believe this sample is adequate because recent reviews have found that most qualitative studies achieve data saturation between 9 and 17 interviews [35]. The semistructured interview guide will contain questions on the existing drivers of poor nurse-client relationships and the contextual factors, barriers, and facilitators that could impact intervention design, implementation, and sustainability. Participants will be recruited through MCH managers. All interviews will be conducted at a convenient location confirmed with the respondents in advance to enable them to identify an alternate location if required. Upon arrival, research assistants will provide detailed information on the study, obtain informed consent, and engage respondents for approximately 45 to 60 minutes in a semistructured audio-taped discussion. The findings will be used in step 2 of the HCD process.

#### Step 2. Consultative Co-design Meetings

In this stage, a transdisciplinary team of purposively sampled MCH nurses and midwives, clients, administrators, and other relevant stakeholders (30 members) will gather to define the challenges based on discovery findings and design an intervention package (prototype) with the highest potential to improve nurse-client relationships considering acceptability and feasibility. Invitation letters will be sent to purposively sampled participants with information on the date of the consultative meeting and preselected venue. This 3-month process will involve 3 consultative meetings: (1) a synthesis meeting to review the qualitative data gathered in step 1 and share insights, experiences, and questions to generate a deeper understanding of the challenges of nurse-client relationships in Shinyanga; (2) an ideation meeting to brainstorm and generate “how might we” questions that facilitate the development of potential ideas for the solution; (3) a prototype and cocreation meeting to evaluate the ideas generated considering pros, cons, and feasibility and develop the initial (rough) prototype model(s) as well as elements crucial to its testing (features, modality, responsible person, etc). Each meeting will be conducted for 4 to 6 hours and all key discussion points will be documented. The findings will inform step 3.

#### Step 3. Validation and Insight Gathering Inquiry

This will involve gathering insights on the rough prototype in Shinyanga MC for 3 months depending on the features of the prototype model to be tested. The aim is to gather qualitative feedback using guided FGDs (6 sessions) with purposively sampled participants to identify features appealing to both nurses and clients for strengthening their relationship to increase MCH service satisfaction, uptake, and continuity. Nurses and clients will be recruited through MCH managers and engaged in 45- to 60-minute audio-taped discussions. The findings will inform step 4.

#### Step 4. Refinement and Adaptation Meeting

The design team will reconvene for 1 day to evaluate the feedback and rough prototype insights as well as to refine and adapt the prototype. Representatives of the participants for rough prototype testing (insight gathering inquiry) will be selected by their peers to join the participants of co-design meetings (40 members) in the refinement and adaptation process leading to the final prototype model. The lessons learned in arriving at the final prototype model will inform step 5.

#### Step 5. Document and Share

The lessons will be synthesized and disseminated to local and international stakeholders. These lessons will form the basis for transitioning the intervention package (prototype) to feasibility and definitive testing.

We will recruit 3 research assistants and train them on the HCD process and techniques pertaining to this study. The discussion, interview, and consultative meeting guides will be pretested in purposefully selected settings and refined to ensure readiness for use in the actual data collection process (Multimedia Appendix 2). Close and supportive supervision of research assistants will be conducted throughout the data collection and analysis stages to ensure data quality.

### Data Management and Analysis

The HCD process will generate data from FGDs, KIIs, and consultative meetings. Data transcription and translation will occur simultaneously by research assistants and verified by the research team. Interview transcripts will be deidentified, pseudonyms generated for each participant, and the data uploaded into the NVivo 12 software (QSR International) for management and deductive thematic coding. A stepwise approach will be used for a deductive thematic analysis of the interview transcripts [36]. First, the research team will examine the research questions and generate several themes based on consensus. This will result in an analytical matrix of the main themes and subthemes. Individual transcripts and phrases (codes) representing participants’ responses to investigators’ questions will be exported to relevant themes and related subthemes within NVivo. A consensus-based approach will then be used by the research team to decide on including codes that do not fit within the developed subthemes and themes; the codes will be excluded if do not provide critical value to the study, as confirmed by subjective and objective evaluations. The data within NVivo will then be exported to Microsoft Word (Microsoft Corporation) for interpretative analysis and report generation.

### Ethics Approval

This study has received ethics clearance from the AKU Ethics Review Committee and The National Institute for Medical Research (NIMR/HQ/R.8a/Vol. IX/3906), as well as local approvals from the Regional Medical Officer of Shinyanga and the Municipal Medical Officer in Shinyanga (Multimedia Appendix 3). At the health facility level, verbal approvals will be sought from the managers of the selected facilities from where nurses and clients will be recruited after providing letters from the district medical officers and copies of ethical clearance. We will ensure responsible conduct of research by obtaining written consent from all participants before participation.

The study does not directly or indirectly expose nurses and clients to any diagnosis or treatment. As safeguards, all study responses will be kept confidential, and data analysis and reporting will be conducted at an aggregated level within the Shinyanga region. Further, all data gathered will not be used for purposes other than the present research. Due to the global COVID-19 pandemic, face masks, sanitizers, and social distancing will be used to mitigate infection transmission during fieldwork activities.

## Results

### Participant Demographics

We will summarize the characteristics of all participants across all stages of the HCD process. Descriptions and tables will be used to present key participant characteristics.

### Findings From the Community-Driven Discovery Inquiry

We expect to generate results from a qualitative study employing FGDs with nurses and MCH clients and KIIs with MCH administrators and stakeholders conducted as part of the community-driven inquiry. The results will include participants’ understanding of what nurse-client relationships mean; their experiences with good and bad nurse-client interactions; the existing drivers of poor nurse-client relationships; and the contextual factors, barriers, and facilitators that could impact intervention design, implementation, and sustainability. The findings will include analysis supported by participant quotes and will form the first set of HCD results to guide the co-design step.

### Findings From Consultative Co-design Meetings

We expect to generate findings from the consultative meeting proceedings conducted as part of the co-design process. These findings will include the discussions with nurses, MCH clients, and key stakeholders in defining the challenges based on discovery findings (synthesis meeting), potential solutions to address the identified challenges (ideation meeting), and the intervention package (prototype and cocreation meeting) with the highest potential to improve nurse-client relationships considering acceptability and feasibility. The findings will be presented through interpretations, participant quotes, tables, and figures and will form the second set of HCD findings and guide the insight gathering/prototype validation step.

### Findings From the Validation/Insight Gathering Inquiry

We expect to subject the emerging prototype to a validation process by gathering insights through FGDs with nurses and clients who were not involved in the initial HCD steps. The results of the FGDs will be analyzed to identify features appealing to both nurses and clients for strengthening their relationship to increase MCH service satisfaction, uptake, and continuity. The findings will be presented through interpretations and the proposed intervention package (prototype). These findings will form the third set of HCD results and will guide the final adaptation step.

### Findings From the Prototype Refinement/Adaptation Meeting

We expect to refine and adapt the prototype based on the insights of nurses and clients who were not part of the initial HCD steps. The findings of the refinement and adaptation meeting will be presented through interpretation, participant quotes, tables, and figures. The final prototype (package of interventions) for strengthening nurse-client relationships in MCH care will also be presented. The findings will guide scholarly discussions and future interventions in a broader setting.

### Dissemination Plan

A number of strategies will be employed to disseminate the results of this interventional study. First, we will employ the AKU networks by sharing a research report with the funding agency (University Research Council in this case), depositing the reports and publications in eCommons, and presenting the findings in AKU-wide forums including journal clubs and research meetings. Second, we will share the results with local nursing and health care authorities by sending summary reports to district and regional medical officers, nursing and midwifery councils, the Ministry of Health, and the National Institute for Medical Research for dissemination through government channels. This will ensure that the proposed interventions contribute to practice, policy, and strategic plan discussions at the local and national levels. We will also present the findings in local health care and scientific forums. We plan to prepare at least 3 research manuscripts to be published in reputed scholarly journals. We will also disseminate the findings at international conferences and share a media brief for the general public.

## Discussion

### Study Overview

The purpose of this study is to pilot an HCD approach for improving provider-client relationships in MCH care in rural Tanzania. Using this approach, the research team seeks to partner with nurses, midwives, clients, and other stakeholders to develop a prototype for addressing the complex problem of nurse-client relationships in Shinyanga.

The principal results are expected to be fourfold. First, the results of the community-driven inquiry including the existing drivers of poor nurse-client relationships and the contextual factors, barriers, and facilitators that could impact intervention design, implementation, and sustainability. These results will be discussed in comparison to previous works that have examined the factors that impact nurse-client relationships in both low- and high-income contexts [10-23]. Second, the results of the consultative meetings conducted as part of the co-design process will be used to design an interventional package (prototype) with the highest potential to improve nurse-client relationships. During this phase, ideation and cocreation meetings will be held to brainstorm and evaluate possible solutions leading to the development of a contextualized rough prototype to address poor nurse-client relationships. The rough prototype may include conventional capacity-building interventions such as training providers on interpersonal communication, the development of an interpersonal encounter algorithm for providers, community sensitization, and advocacy activities on client charters, and competitions to incentivize good provider behavior such as the “nurse of the month/year” duly informed by formative research. It is also possible that the capacity-building and community mobilization and advocacy activities will leverage technology (mobile health) using provider algorithms to remind nurses and midwives to conduct themselves in a certain way, and to develop behavior change and communication strategies for clients through SMS text messaging or audiovisual methods. The emerging rough intervention package will be discussed in view of previous interventions for strengthening nurse-client relationships [19,37-42]. Third, the results of prototype validation will be used to identify features appealing to both nurses and clients for strengthening their relationship to increase MCH service satisfaction, uptake, and continuity. We will gather feedback on the rough prototype for refining it accordingly. A preliminary theory of change map will be developed based on the stakeholder consultations. The results of the prototype validation will be discussed considering acceptable and feasible interventions that have been proposed to improve nurse-client relationships [19,37-42]. Lastly, we will evaluate the results of the refinement and adaptation meeting leading to a final prototype for strengthening nurse-client relationships. We will examine the existing body of literature in both high- and low-income countries to assess the novelty of the emerging prototype, whether it has been considered in other settings, and key considerations for implementation. We will consider the body of literature on interventions aimed at strengthening both nurse-client relationships as well as general provider-client relationships [19,23,37-42]. Intrinsic to the co-design process is the fact that stakeholders jointly understand a problem, act on it, and learn from working collaboratively to contest power relations and effect change [26-32].

As noted above, the strength of the HCD methodology is that it is a highly adaptive and creative approach to problem-solving [26-32] and will enable the team to deeply understand the drivers of poor client-provider relationships and ensure a collaborative approach to the design of solutions by stakeholders, yielding a final model that is highly feasible as a result. We will document the co-design process and develop the final prototype manual and associated materials to facilitate replication of the intervention in similar or other settings. Specifically, we envision applying for additional funds to test the emerging prototype within Tanzania and across the East African region to determine whether it could be applicable to a much broader African context.

### Limitations

The application of HCD to develop a prototype for improving nurse-client relationships is not without limitations. The HCD intervention uses nurses as exemplars of providers to codevelop a prototype for strengthening interpersonal relationships in MCH care in a rural setting. However, patients interact with multidisciplinary teams of providers within health care settings. Conducting a similar study with other providers such as doctors and in a different setting may yield a different prototype. However, this being the first study in this context, future inquiry may extend beyond the nursing profession and rural context.

### Comparison With Prior Work

Discussion of the emerging findings will be contextualized based on previous studies and interventions on strengthening provider-client relationships in Tanzania and beyond [17-25,37-41]. In particular, the results will be discussed taking stock of a previous study in a similar setting conducted by the principal investigator that proposed the need for novel approaches to address the complexity of patient-provider relationships [23].

### Conclusions and Future Directions

In conclusion, the HCD approach may provide a novel entry point for strengthening provider-client relationships where clients are invited to partner with providers in the design of highly acceptable and feasible interventions. The results of this pilot study will inform the design of interventions and policies to strengthen interpersonal relationships in health care settings more broadly. Moreover, future implementation teams and researchers can learn from the experience of this HCD intervention to guide program development.

## Appendix

Multimedia Appendix 1 Human-centered design (HCD) steps, population, activities, and sample size.

Multimedia Appendix 2 Copies of data collection tools.

Multimedia Appendix 3 Ethical clearance certificate from the National Institute for Medical Research, Tanzania.

## Abbreviations

AKU: Aga Khan University
DC: District Council
FGD: focus group discussion
HCD: human-centered design
KII: key informant interview
MC: Municipal Council
MCH: maternal and child health

## Data Availability

The authors confirm that the data supporting the findings from the HCD intervention will be available in future publications as supplementary materials. Additional data on the HCD process that are not part of the published article will be available on request from the AKU through the corresponding author (KI). Some data may not publicly available for ethical reasons (ie, information that could compromise the privacy of research participants).
