# Supplementary material for: Strengthening Interpersonal Relationships in Maternal and Child Health Care in Rural Tanzania: Protocol for a Human-Centered Design Intervention
Source: JMIR Res Protoc. 2022 Jul 7;11(7):e37947. doi: 10.2196/37947 (PMC9305451; doi:10.2196/37947)
Supplement: Multimedia Appendix 1 [file resprot_v11i7e37947_app1.docx]

**Appendix 1: HCD Steps, population, activities and sample size**

| **Step** | **Activity/ Study** | **Methods** | **Sample size** | **Population** |
| --- | --- | --- | --- | --- |
| **Step 1: Discovery** | Community discovery study | Qualitative | 8 FGDs  10 KIIs | 4 FGDs with Nurses and midwives, 4 FGDs with women attending MCH, and 10 KIIs with MCH administrators |
| **Step 2: Co-Creation** | Synthesis meeting | Meeting | 30 participants | 10 nurses and midwives, 10 clients and 10 administrators, researchers and other stakeholders |
|  | Ideation | Meeting | 30 participants | 10 nurses and midwives, 10 clients and 10 administrators, researchers and other stakeholders |
|  | Prototyping and creation of intervention package | Meeting | 30 participants | 10 nurses and midwives, 10 clients and 10 administrators, researchers and other stakeholders |
| **Step 3: Validation** | Rough prototype insight gathering | Qualitative | 6 FGDs | 3 FGDs with nurses and midwives, and 3 FGDs with clients in selected areas of Shinyanga  *Note: Feedback collected through qualitative inquiry* |
| Step 4: Refine/ Adapt | Refinement and adaptation of prototype | Meeting | 30 +10 participants | 10 nurses and midwives, 10 clients and 10 administrators, researchers and other stakeholders  10 representatives of rough prototype insight gathering inquiry |
| Step 5: Document/ Share | Manuscript development | Qualitative | 3 manuscripts | Design process; discovery findings and outcome of the intervention |
