# Supplementary material for: Strengthening Interpersonal Relationships in Maternal and Child Health Care in Rural Tanzania: Protocol for a Human-Centered Design Intervention
Source: JMIR Res Protoc. 2022 Jul 7;11(7):e37947. doi: 10.2196/37947 (PMC9305451; doi:10.2196/37947)
Supplement: Multimedia Appendix 2 [file resprot_v11i7e37947_app2.pdf]

AGA KHAN UNIVERSITY
SCHOOL OF NURSING AND MIDWIFERY
INTERVIEW GUIDES

COMMUNITY DISCOVERY INQURY

PROJECT TITLE: IMPROVING NURSE- CLIENT RELATIONSHIPS IN MATERNAL AND CHILD HEALTH CARE IN RURAL TANZANIA: A HUMAN CENTERED DESIGN (HCD) APPROACH

PART 1: FOCUS GROUP DICUSSION WITH NURSES AND CLIENTS

- *Ensure availability of quit and safe venue*
- *Welcome all the participants*
- *Read the consent form*

1: PARTICIPANTS DEMOGRAPHIC INFORMATION

PARTICIPANT	AGE	GENDER	MARITAL STATUS	LEVEL OF LICENCE (NURSES ONLY)	HIGHEST LEVEL OF EDUCATION	LEVEL OF HEALTH FACILITY	YEARS OF MCH CARE EXPERIENCE
1							
2							
3							
4							
5							
6							
7							
8							

Facilitator____________________

Notetaker____________________

Starting time_________________

2: FGD QUESTIONS

1. What does a good nurse-client relationship mean to you? (*Probe: ever experienced good relationship with nurse/clients? What happened?)*
2. What does a bad/poor nurse-client relationship mean to you? (*Probe: ever experienced bad relationship with nurse/clients? What happened?)*
3. What are the benefits of having a good relationship with your nurse/client in MCH care?
4. What are the consequences of having a bad relationship with your nurse/client in MCH care? *(Probe: consequence to nurses, consequence to clients, consequence to health system?*
5. What are the contributors to a bad relationship between nurses and their clients in MCH care in Shinyanga? *(Probe: Nurse factors, client factors, health system factors?)*
6. What are the existing strategies for strengthening nurse-client relationships in MCH care in Shinyanga?
7. What are your recommendations (strategies) for strengthening relationship between nurse and their clients within MCH care in Shinyanga? *(Probe: How each strategy mentioned can be made much more successful? Key considerations when implementing each strategy? What are the possible barriers that can impact the successful implementation of each strategy mentioned?)*
8. Any other comment in relation to nurse-client relationship in Shinyanga?

THANK YOU   End Time______________________

AGA KHAN UNIVERSITY
SCHOOL OF NURSING AND MIDWIFERY
INTERVIEW GUIDES

PART 2: KII WITH MCH ADMINISTRATORS

- *Ensure availability of quit and safe venue*
- *Welcome the participant*
- *Read the consent form*

Interviewer ___________________
Starting time__________________

1: PARTICIPANTS DEMOGRAPHIC INFORMATION

1. Participant title: ______________________
2. Age__________________________________
3. Gender______________________________
4. Highest level of Education____________
5. Marital Status________________________
6. Years of MCH leadership______________

2: KII QUESTIONS

1. What are your roles and responsibilities in relation to MCH care?
2. What does a good nurse-client relationship mean to you? (*Probe: ever received clients complements of nurses? What happened?)*
3. What does a bad/poor nurse-client relationship mean to you? (*Probe: ever handled client's complaints about nurses? What happened?)*
4. What are the benefits of having a good relationship with your nurse/client in MCH care?
5. How a bad relationship between nurses and clients have impacted MCH care in Shinyanga? *(Probe: consequence to nurses, consequence to clients, consequence to health system?*
6. What are the contributors to a bad relationship between nurses and their clients in MCH care in Shinyanga? *(Probe: Nurse factors, client factors, health system factors?)*
7. What are the existing strategies employed for strengthening nurse-client relationships in MCH care in Shinyanga?
8. What are your recommendations (strategies) for strengthening relationship between nurse and their clients within MCH care in Shinyanga? *(Probe: How each strategy mentioned can be made much more successful? Key considerations when implementing each strategy? What are the possible barriers that can impact the successful implementation of each strategy mentioned?)*
9. Any other comment in relation to nurse-client relationship in Shinyanga?

THANK YOU End Time,_______________________

AGA KHAN UNIVERSITY
SCHOOL OF NURSING AND MIDWIFERY
INTERVIEW GUIDES

2. CO-DESIGN MEETINGS AGENDA

PROJECT TITLE: IMPROVING NURSE- CLIENT RELATIONSHIPS IN MATERNAL AND CHILD HEALTH CARE IN RURAL TANZANIA: A HUMAN CENTERED DESIGN (HCD) APPROACH

MEETING OBJECTIVES

1. Synthesis meeting to review the qualitative data gathered in step 1, share insights, experiences and questions to generate deeper understanding of challenges of nurse-client relationships in Shinyanga;
2. Ideation meeting to brainstorm and generate 'how might we" questions that facilitate development of potential ideas for the solution;
3. Prototype meetings and co-creation meeting to evaluate the ideas generated considering pros, cons and feasibility and to develop initial (rough) prototype model (s) as well as elements crucial to its testing (features, modality, responsible person etc.).

PARTICIPANTS: Nurses currently working in MCH (at least one year), women currently attending to MCH clinics (at least three visits in a year) and MCH stakeholders in Shinyanga

DRAFT MEETING AGENDA

TIME	SYTHESIS MEETING	IDEATION	PROTOTYPE AND C0-CREATION
08:00-09:00	REGISTRATION	REGISTRATION	REGISTRATION
09:00-09:30	INTRODUCTION TO HCD STUDY	KEY FINDINGS FROM SYNTHESIS MEETING	KEY FINDINGS FROM IDEATION MEETING
09:30-10:00	KEY FINDINGS FROM INQUIRY STUDY	GROUP WORK	GROUP WORK
10:00-10:30	BREAKFAST	BREAKFAST	BREAKFAST
10:30-12:30	GROUP WORK	GROUP WORK	GROUP PRESENTATIONS
12:30-13:00	LUNCH	LUNCH	LUNCH
13:00-14:00	GROUP PRESENTATIONS	GROUP PRESENTATIONS	DISCUSSION ON THE PROTOTYPE
14:00-14:30	CLOSING	CLOSING	CLOSING

AGA KHAN UNIVERSITY
SCHOOL OF NURSING AND MIDWIFERY
INTERVIEW GUIDES

3. PROTOTYPE VALIDATION

PROJECT TITLE: IMPROVING NURSE- CLIENT RELATIONSHIPS IN MATERNAL AND CHILD HEALTH CARE IN RURAL TANZANIA: A HUMAN CENTERED DESIGN (HCD) APPROACH

FOCUS GROUP DICUSSION WITH NURSES AND CLIENTS

- *Ensure availability of quit and safe venue*
- *Welcome all the participants*
- *Read the consent form*

1: PARTICIPANTS DEMOGRAPHIC INFORMATION

PARTICIPANT	AGE	GENDER	MARITAL STATUS	LEVEL OF LICENCE (NURSES ONLY)	HIGHEST LEVEL OF EDUCATION	LEVEL OF HEALTH FACILITY	YEARS OF MCH CARE EXPERIENCE
1							
2							
3							
4							
5							
6							
7							
8							

Facilitator____________________
Notetaker____________________
Starting time_________________

2: FGD QUESTIONS (MAY BE ADAPTED BASED ON THE PROTOTYPE DEVELOPED)

CONTEXTUAL BACKGROUND (READ)

We conducted research in Shinyanga to examine the drivers of poor nurse-client relationships in MCH care and possible solutions. We then brought together a group of nurses, clients and other MCH stakeholders and developed an intervention package for improving nurse-clients relationship. The intervention package includes____

1. What do you think about this intervention package? *(Probe: do you think they can improve the relationship between you and your clients/nurses in MCH care? If yes, why? If no, why not?)*
2. What is features of this intervention appeals most to you? What features do not appeal to you?
3. What was overlooked when developing these interventions?
4. What are the possible barriers for implementing these interventions?
5. What modifications do you suggest for this interventions package? *(Probe for suggestions for each proposed activity)*
6. Any other suggestion?

THANK YOU End Time______________________

AGA KHAN UNIVERSITY
SCHOOL OF NURSING AND MIDWIFERY
INTERVIEW GUIDES

4. REFINEMENT/ADAPTATION MEETING

PROJECT TITLE: IMPROVING NURSE- CLIENT RELATIONSHIPS IN MATERNAL AND CHILD HEALTH CARE IN RURAL TANZANIA: A HUMAN CENTERED DESIGN (HCD) APPROACH

MEETING OBJECTIVE: Evaluating the feedback and rough prototype validation insights as well as refine and adapt the prototype.

PARTICIPANTS: Nurses currently working in MCH (at least one year), women currently attending to MCH clinics (at least three visits in a year) and MCH stakeholders in Shinyanga

DRAFT MEETING AGENDA

TIME	ACTIVITY
08:00-09:00	REGISTRATION
09:00-09:30	KEY FINDINGS FROM VALIDATION INQUIRY STUDY
09:30-10:30	GROUP WORK
10:30-11:00	BREAKFAST
11:00-12:30	GROUP WORK
12:30-13:00	LUNCH
13:00-14:00	GROUP PRESENTATIONS
14:00-14:30	DISCUSSION ON FINAL PROTOTYPE
14:30-15:00	CLOSING
