# Supplementary material for: Strengthening Interpersonal Relationships in Maternal and Child Health Care in Rural Tanzania: Protocol for a Human-Centered Design Intervention
Source: JMIR Res Protoc. 2022 Jul 7;11(7):e37947. doi: 10.2196/37947 (PMC9305451; doi:10.2196/37947)
Supplement: Multimedia Appendix 3 [file resprot_v11i7e37947_app3.pdf]

THE UNITED REPUBLIC OF TANZANIA

National Institute for Medical Research
3 Barack Obama Drive
P.O. Box 9653
11101 Dar es Salaam
Tel: 255 22 2121400
Fax: 255 22 2121360
E-mail: ethics@nimr.or.tz

Permanent Secretary
Ministry of Health
Government City Mtumba, Health Road
P.O. Box 743
40478 Dodoma

NIMR/HQ/R.8a/Vol. IX/3906

04th February 2022

Dr. Kahabi Isangula
Assistant Professor
Aga Khan University
School of Nursing and Midwifery
P O Box 38129
Dar es Salaam

RE: ETHICAL CLEARANCE CERTIFICATE FOR CONDUCTING MEDICAL RESEARCH IN TANZANIA

This is to certify that the research entitled: **Improving nurse-client relationships in maternal and child health care in rural Tanzania: A human centred design (HCD) approach (Isangula K. et al.)** has been granted ethical clearance to be conducted in Tanzania.

The Principal Investigator and supervisor of the study must ensure that the following conditions are fulfilled:

1. Progress report is submitted to the Ministry of Health and the National Institute for Medical Research, Regional and District Medical Officers after every six months.
2. Permission to publish the results is obtained from National Institute for Medical Research.
3. Copies of final publications are made available to the Ministry of Health and the National Institute for Medical Research.
4. Any researcher, who contravenes or fails to comply with these conditions, shall be guilty of an offence and shall be liable on conviction to a fine as per NIMR Act No. 23 of 1979, PART III Section 10(2).
5. Sites: Shinyanga region.

Approval is valid for one year: 04th February 2022 to 03rd February 2023.

Name: Prof. Yunus Daud Mgaya

Signature
CHAIRPERSON
MEDICAL RESEARCH
COORDINATING COMMITTEE

Name: Dr. Aifello Wedson Sichalwe

Signature
CHIEF MEDICAL OFFICER
MINISTRY OF HEALTH

CC: Director, Health Services-TAMISEMI, Dodoma.
RMO of Shinyanga region.
DMO/DED of respective districts.
